# “We Are Good Neighbors, But We Are Not Carers!”: Lived Experiences of Conflicting (In)dependence Needs in Retirement Villages Across the United Kingdom and Australia

**DOI:** 10.1093/geront/gnab164

**Published:** 2021-11-03

**Authors:** Sam Carr, Chao Fang

**Affiliations:** Department of Education and Centre for Death and Society, University of Bath, Bath, UK; Department of Education and Centre for Death and Society, University of Bath, Bath, UK

**Keywords:** Ageism, Othering, Qualitative, Retirement living

## Abstract

**Background and Objectives:**

This study sought to qualitatively explore the lived experiences of 80 older people living in retirement villages across the United Kingdom and Australia. We focused on residents’ narratives around the themes of independence/dependence.

**Research Design and Methods:**

Qualitative semistructured interviews permitted in-depth exploration of how older people understood and experienced issues related to independence/dependence in the context of retirement living.

**Results:**

Core themes identified strikingly different and often competing needs and narratives around independence/dependence. Of note was the fact that narratives and needs around independence/dependence frequently collided and conflicted, creating a sense of “us” and “them” in the retirement community. The primary source of such conflict was reflected by the fact that residents seeking a “prolonged midlife” often felt that frailer and more dependent residents were a burden on them and were not suited to an “independent living community.”

**Discussion and Implications:**

Our findings are discussed in relation to the challenges such competing narratives create for retirement villages as living environments for a group of people who are far from homogenous.

It has been estimated that 3% of Australians older than 65 years reside in retirement communities (Kennedy & Coates, 2008) and between 7% and 17% in the United States (Omoto & Aldrich, 2006). The number in the United Kingdom is estimated to be much lower (0.6%), but there is a growing demand for retirement communities in the country’s housing market (Associated Retirement Community Operators, 2018). Glass and Skinner (2013) have highlighted that little attention has been devoted to the meaning of terms such as “retirement community,” “retirement village,” or “independent-living retirement community,” and such labels have been broadly applied to a variety of housing options for older people.

Kingston et al. (2001) outlined that there are some basic definitional characteristics that such communities tend to share: (a) residents who are no longer in full-time employment, (b) an age-specific population living in the same bounded geographic area, (c) some degree of collectivity that may include shared interests, activities, or facilities, and (d) some sense of autonomy and security. However, beyond these basic parameters, retirement communities can vary considerably in relation to an array of factors such as the provision of assisted living or continued care on-site, a managed transition from independent living to care-home facilities, shared mealtimes, and housekeeping and domestic assistance (Glass & Skinner, 2013).

Research has identified various benefits to retirement living communities for older people, including enhanced social connection, emotional security, and retention of a physically active lifestyle (Jenkins et al., 2002; Schwitter, 2020). Downsides have also been identified, including confusion, depression, and anxiety associated with the transition from, and sense of loss of valued former lives, particularly in those who involuntarily move to a retirement community (Bekhet et al., 2009). Additionally, division and tension between residents within retirement communities are not uncommon (Shippee, 2012). In this study, we focused on experiences of conflict and tension in relation to the contrasting needs of residents in “independent-living retirement villages,” purpose-built, geographically bounded villages for the older than 55 years group, offering on-site shared leisure activities and facilities, and access to an “independent lifestyle” (we describe the villages in more depth in the *Method* section).

## Contrasting Needs in Retirement Villages

Wiles et al. (2012) argued that “[b]y treating place as a mere ‘container’ and ‘older people’ as a homogenous category, there can be inadequate recognition of diverse needs” (p. 358). Bekhet et al.’s (2009) exploration of the reasons older people moved to a retirement community identified a diverse array of motives labeled “pull” (e.g., the community was closer to family, offered enhanced security, the prospect of enhanced activity, or perceived retention of independence) and “push” (e.g., loneliness, failing health for themselves or a spouse, needing more help) factors. Furthermore, they identified that the reasons people chose to relocate were typically diverse, frequently contrasting and conflicting, and often reflected a combination of “pull” and “push” factors.

Research has also identified significant diversity in relation to the meaning and experience of independence for older people (Bekhet et al., 2009; Hillcoat-Nalletamby, 2014; Shippee, 2012). Hillcoat-Nallétamby’s (2014) qualitative exploration of older people’s understanding and needs in relation to independence and autonomy across a range of living settings revealed distinct dimensions such as executional autonomy (e.g., executing tasks, to varying degrees, alone and without help), decisional autonomy (e.g., making decisions about oneself and for oneself), spatial independence (having a private, personal living space), and social independence (the freedom to socialize or not and to decide who one socializes with and when). Such distinct dimensions of autonomy and independence highlight the diverse ways in which older people may look for and experience autonomy and independence and reinforce the argument that autonomy and independence should not be thought of as unidimensional constructs.

Hillcoat-Nallétamby (2014) identified that some individuals wished to,

act as independent, self-sufficient agents, with strong authorial control over their choices and actions. In this sense, people explicitly reject support or do not ask for help, sometimes through fear that accepting it will signal loss of ability to live independently or because it evokes a sense of reduced self-determination and control. (p. 427)

In contrast, others sought to maintain a similar sense of authorial control over their lives yet preferred to exercise the choice to request extra-care as deemed necessary, because they felt able to acknowledge a need for assistance in certain aspects of their lives. Clearly, what dependence and independence *mean* for older people varies and may be connected to their identity in a broader sense.

Despite being an age-segregated housing model for older people, retirement villages are home to considerably diverse residents of a wide age range, experiencing significantly different health/cognitive challenges, from varied backgrounds, with different ideas about dependence and/or independence, and different reasons for moving to a retirement community. Nonetheless, this diverse group of older people often live alongside each other and are part of the same “retirement community.”

## The Current Study

The current study draws upon a large qualitative study of 80 older people residing in independent living retirement villages across the United Kingdom and Australia. We sought to explore lived experiences of older people in relation to their motives and needs around independence/dependence, and how these motives and needs aligned or collided with those of other residents. There is a longstanding debate around “otherness” and the “othering” of older people in contemporary society (van Dyk, 2016). Jönson (2013) has discussed how such othering can often be identified by listening carefully to language and narrative in relation to how older people are talked about—by themselves and others. Particular attention was therefore paid to divisions made between “us” (as a distinct group of residents, with similar values and/or needs) and “them” (as a separate group, with competing or conflicting needs) within the villages we investigated, and how such divisions reflected or shaped a sense of otherness that was intimately connected to the meaning of retirement living for older people (Shippee, 2012). It is important for policymakers and developers to better understand how retirement villages emerge and are experienced in relation to a diverse range of competing, contrasting, and conflicting needs and motives. Such understanding will be of importance in relation to better accommodating, integrating, and understanding the diverse and/or competing needs of residents within such villages.

## Method

### Sample

A total of 80 interviews were conducted in eight retirement villages across the United Kingdom and Australia. Four villages were selected from one operating company in each country, and the selection was based on consultation with the company and the village senior management teams. Management teams considered which villages they felt were most representative in terms of gender, age, and ethnicity. All these villages were independent-living retirement villages where residents had to be older than 55 years and have no dependent care needs (although the definition of dependency was often vague). These villages consisted of a mixture of houses and flats that could be purchased or rented. All residents had access to community-based social activities and were within walking distance of community-based and often geographically centralized amenities (e.g., restaurants, bars/pubs, gyms/pools, libraries, and maintenance offices). While prioritizing independent living, these villages also offered short-term domiciliary care (e.g., cleaning, cooking). Those with long-term care needs (again loosely defined) would be required to move to more advanced assisted-living facilities. The village size varied between 50 and 150 residents at the time of interviews.

The UK sample consisted of 40 participants in the North-West, South-West, South-East, and Midlands, with an average residence of 2.8 years. The Australian sample was collected from 40 participants in a metropolitan area of Southern Australia, with an average residence of 8 years. All participants were 55 years or older with an average age of 79 (*SD* = 7.6). The oldest participant was 93 and the youngest was 55, and the gender split was 55 women and 25 men. Despite the wide age range, none of the participants were facing any severe or progressively life-threatening illnesses at the time of the interview, although a few had chronic health conditions. Twenty-six people were married and were living with their spouse, while the remaining 54 participants were living alone due to widow(er)hood, divorce, or unmarried status. More than half of the participants (*n* = 45) had lost loved ones (often a spouse or partner) in recent years.

Participants were recruited through village managers who acted as gatekeepers to introduce the research project and researchers to the residents. The support of the village managers fostered a clear sense of trust between participants and the researchers that persisted through the research process. With this rapport, the interviewers aimed to be “a safe, interested stranger,” with whom the participants felt at ease to share their often painful and previously unspoken life experiences (Johnson, 2013, p. 182). Residents were informed by village managers that the study sought to talk deeply to older people about their inner emotional lives, feelings of connection and disconnection, relationships, life history, and lived experiences of retirement living. Residents then contacted village managers if they felt that they wished to take part. Subsequently, a team of four trained researchers (including the authors) in the United Kingdom and four trained researchers in Australia contacted these residents by telephone.

The study was approved by the Research Ethics Committee at the authors’ institution. Ethical approval was also granted according to the internal processes in place for the management teams of villages in both the United Kingdom and Australia. Furthermore, permission was also gained from the local site managers at each of the eight participating villages.

### Interviews

All interviews were conducted in the participants’ homes independently by the eight researchers between October 2019 and February 2020 in the United Kingdom and Australia. Interviews ranged from 70 to 200 min in length and averaged around 100 min. Participants were alone with the interviewer for all interviews. Where a spouse or partner was at home at the time of the interview, they were not present in the room. The project generated approximately 8,000 min of in-depth data that were audio-recorded and professionally transcribed.

The data presented in this article were part of a broader in-depth qualitative listening exercise. Our objective with the interviews was to allow participants the time and space to talk freely about their thoughts and feelings in relation to a number of key areas: (a) their lives up to this point (including childhood, adolescence, career, family, and anything else they wished to share and discuss), (b) their closest relationships, (c) experiences of loss, (d) feelings of loneliness and isolation, (e) their decision to move to retirement living, and (f) their lived experiences of the retirement community. Each of these areas was broad, complex, overlapped with other areas, and opened-up numerous avenues of discussion that were personal and unique to the person concerned and their lived experiences. It should be noted that this article deals specifically with older people’s decision to move to retirement villages and their lived experiences of these villages. However, this is not to say that other parts of the interview/s were not relevant or important in relation to this focus—so all parts of the full interview formed a part of our analysis. We adopted an approach to our interviews (Kvale & Brinkmann, 2009; Morgan & Burholt, 2020) that enabled participants to construct their stories around the core areas of the interview schedule. Participants could talk as freely as they wished, and interviewers were trained to listen and to interrupt minimally. Several participants commented on the value of the interview for them—it provided a welcome and (often) rare space for them to open up and feel genuinely “listened to.”

### Data Analysis

A thematic analysis (Braun & Clarke, 2012) was conducted to interpret these rich data and an inductive approach was adopted. This method of analysis allowed for important messages about lived experiences and nuanced feelings in relation to retirement community living to emerge from a large amount of data, without being dominated by preexisting frameworks (Mason, 2002). In so doing, a rich understanding of how participants experienced retirement community living in relation to core issues such as independence, dependence, and division arose from the analysis.

**Figure 1.**
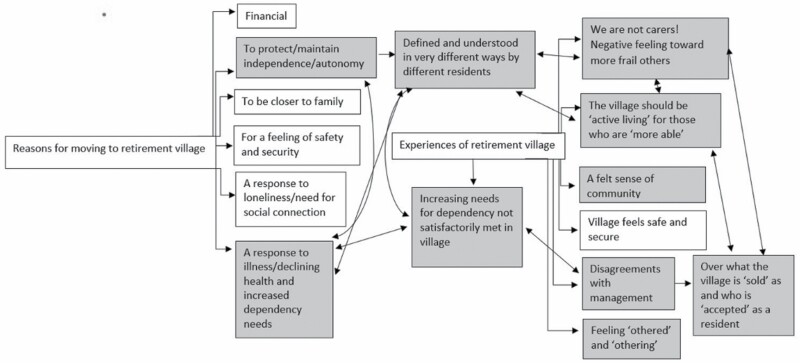
An illustration of an early iteration in our coding process. Shaded boxes reflect codes that we identified as particularly relevant to the research questions for this study. Codes we initially identified as interconnected are joined by arrows.

**Figure 2.**
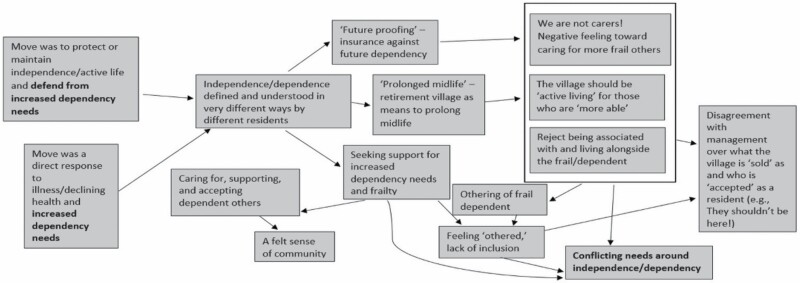
An illustration of a later iteration of our coding process that reflects refinement and elaboration of the initial codes identified in Figure 1. Here, we refine our codes related to independence/dependence/autonomy.

The analysis was conducted by both authors, who read the interview transcripts and notes thoroughly before conducting independent coding of each interview transcript. A combination of NVivo 12, a qualitative analysis software package, and more traditional Microsoft Word-based reading and coding was used to manage and analyze the large data set. We followed the six-phase thematic analysis process recommended by Braun and Clarke (2012), which involved familiarizing ourselves with the data, generating initial codes, searching for themes, reviewing and discussing potential themes, refining and naming themes, and writing themes up. The two authors met frequently to discuss and compare findings and codes. If there was disagreement or divergence on codes, further reading and discussion were conducted until a consensus was met. Upon the completion of coding, final codes were converted to a diagrammatic representation. Figures 1 and 2 display an early and later iteration of our coding processes in diagram form.

## Findings

Approximately 15% (*n* = 12) of interviewees (the “prompted by dependency group”) revealed that they had made the decision to move to a retirement community for more concentrated social and practical support *following* an experience that brought about an increased sense of dependency for either them or their spouse. This typically involved either the loss of a spouse and/or significant physical or cognitive health deterioration for themselves or their spouse that prompted them to seek a more assistive, secure, and supportive living environment. In contrast, 50% (*n* = 40) of interviewees (the “predependency group”) told us that they had made the move to retirement living *before* experiencing any sort of significant loss or increase in dependency whatsoever. Typically, people in this group told us that their main motivation for moving to the retirement community was to access social interactions and purpose-designed facilities that can help them either to (a) facilitate “active living” as they aged and/or (b) to ensure they would be ready and prepared for increased dependency in the future. Finally, approximately 35% (*n* = 28) of interviewees (the “became-more-dependent-in-place group”) revealed that they had initially moved to the retirement community for the same reasons as the “predependent” group but had experienced increasing dependency needs at some point since moving.

It was surprising to see the diverse and often competing needs of these participants regardless of them living in largely similar independent-living villages. While acknowledging that these self-selected participants’ accounts were potentially biased due to their special conditions and needs, we were able to use their unique perspectives to capture their diverse physical, cognitive, relational, social, and emotional needs and experiences. These include the varied ways they experienced and relied upon the retirement community; the need for independence was interpreted and negotiated by these residents in strikingly different and frequently competing manners. We present our findings in relation to this issue according to some primary themes: (a) remaining “independent and active,” (b) coping with increasing dependency needs, (c) conflicting needs around independence and dependence, and (d) developing a sense of community (Figure 1). Themes 1 and 2 provide the background of the diverse groups of residents with different needs and expectations. Theme 3 highlights the primary findings of this study on the significant differences and conflicts in relation to these diverse older people’s desires from their retirement living. Theme 4 reveals the importance of “aging together” as a means of alleviating conflict within the villages. To protect participants’ confidentiality, pseudonyms are used throughout.

### Remaining Independent and Active

#### Prolonging midlife

The retirement villages in our study were often branded by the organizations that developed and managed them as “older than 55 years active independent living” villages. For many participants (about 25%), typically those from the predependency group described above, comparatively younger, newly moved (within the last 2 years), and who still considered themselves to be “physically active,” the move to a retirement community was designed to help maintain a feeling of independence and “active living” and essentially to prolong midlife.

So, then this decision to move. So, then I thought okay, how can I be independent? So rather than just going to another retirement home …. Although it was an independent flat the set-up was a lounge with the chairs all around [laughs]. I’m thinking, forget it [laughs]. And I appreciate I’m a young 64-year-old and so I decided this concept of village, what does it mean, to keep me connected, not become isolated on my own. But also leave me my independence. I’m very interested in the environment and stuff like that. (Lucy, 64, UK)

Even for those (10%) like Steve who was relatively older, retirement living was seen as a means of maximizing a healthy and fun lifestyle.

Well, if I was 30 again, I’d still be playing squash and tennis and I’d be running and doing all these things, but you’re not, so you can’t do that. Yes, I think we should set out to live life to the full and a place like this gives you the opportunity to continue to do that in your more mature years. (Steve, 81, UK)

#### Future proofing

This prolongation of midlife was often accompanied by an awareness that increasing dependency may be a future challenge, for which many people (30%), exclusively from the predependency group, wanted to feel “prepared” and “ready” but that was not yet a reality. David had moved to the retirement community with his wife 3 years ago and clearly articulated this position:

I think we are very fortunate. We are still reasonably fit. We’ve got a strong stable background of family and friends. This is a place which hopefully will be easy to live and do the things we want to and keep fit and healthy.

We only wanted to move once. So, you want to be somewhere where you’re both comfortable and ready for the future, that was certainly part of it, yes. (David, 76, UK)

Paula had moved to the retirement village within the last year. She expressed similar sentiments.

As a widow. Yes. It’s a kind of “future proofing” exercise because you have got the buses when you can’t drive anymore, you can get out to get your shopping easily, you are not going to be marooned, plus there’s all these facilities … and if you want to go out to play, they’ve got something going on every day, all day long [laughs]! (Paula, 72, UK)

Roger had moved to the village with his wife, Margaret, and told us that the village reflected a sort of safety mechanism designed to alleviate future loss and dependency.

That’s another potential reason for moving somewhere like this, possibly sooner than you really need to, because if Margaret did die before me, in the future, there are a lot of people I know around here. I wouldn’t be particularly lonely. I would miss Margaret a hell of a lot, but I could exist, survive. (Roger, 73, UK)

#### Rejecting being associated with the “old” and “dependent”

In some residents (15%), from the predependency group, the desire to hold on to active, independent living was also accompanied by a rejection of the stereotypical idea of retirement villages and a differentiation between themselves and concepts related to “frailty,” “dependency,” or “being old.” Polly expressed a desire to distance herself and the community she lived in with what she felt was a negative stereotype of a retirement community:

I saw the look of horror on people’s face when you say where you are going to is a retirement village. You are consigning yourself to the rubbish tip, being put in a corner to rot. Those places I saw when I was looking for my mother, which smelt of cabbage and urine, and people were in chairs in a canteen to eat food which was overcooked and under-loved, the way that there was nowhere for them to sit except in a circle with the television blaring—that is absolutely NOT what this [village] is. (Polly, 73, UK)

Some predependent residents (10%), like Jane, however, also expressed objections against being surrounded by “older” people.

The older people make you feel older. Yes. They can’t do as much …. We do help them, but we can’t be living our life around them. (Jane, 72, UK)

### Coping With Increasing Dependency Needs

A smaller subset (15%) of people was prompted to move into retirement villages by direct experiences of increasing dependency needs, typically related to their health or bereavement. They thus had direct intentions to find both social and practical support from the retirement community to better cope with these challenges while maintaining independence to some extent. Peter had recently moved into a UK retirement community with his wife, Sue. The couple had increasingly been struggling with Sue’s deteriorating dementia and viewed the village as a solution to some of the challenges they faced.

Well, it all stems really from Sue’s illness … the problems that have occurred, and we thought this [village] would be the answer …. I was under the impression that’s what we would find by moving here. (Peter, 78, UK)

Patricia sought to restore a sense of independence by moving to the retirement community following a major hip operation that left her debilitated and feeling increasingly isolated.

I thought, this won’t do at all. I am an independent person. I do not like this, being trapped like this … it seemed to me that it was time I was thinking about finding somewhere like this [village] to move to—because another winter like that and I would have got so depressed I would probably have done myself in. (Patricia, 85, UK)

Furthermore, for some residents (10%), moving into retirement villages had not only helped them to cope with increasing dependency needs but had also contributed to restoring a sense of safety, security, and autonomy. Meg told us:

I feel as though with this organisation [village provider], I feel cared for. I feel safe and cared for and for me, that’s enough—to feel safe and cared for, right. The rest is up to me now and I’m doing the best I can. (Meg, 84, Australia)

### Conflicting Needs Within the Village

#### “We are not carers!”

Perhaps the *most powerful* theme to emerge from our data set (in the sense that it reflected how the above needs of the different groups were interrelated and experienced in relation to others in the community setting) was a conflict in relation to how people with different needs and ideas around independence and/or dependence experienced “othering” when living alongside each other in retirement villages (Shippee, 2012). Frequently, those who formed part of the “predependency group” (30%) felt challenged by, resentful of, or objected to the presence of other residents who were more frail, dependent, or older.

I mean, the average age here is nearly 80. That’s not “active retired,” is it? Somebody moved in who was 94?! (Ralph, 72, UK)

No, because they shouldn’t be here! They should go into a care home. We’re not carers! We’re not a care home! This is for over 55s, actives, active living. Now, people if they buy down here and they’re in a wheelchair, it’s up to them, they’ve got to work it out, how to get to the club house. So, you’ve got to be active, otherwise there’s a care home for you. (Margaret, 78, UK)

I don’t think the people [here] are vetted enough. I think the main criteria is you’ve got the money. I don’t necessarily think there ought to be MORE support—I think there ought to be LESS people who require support here. (Paul, 74, UK)

Some people (15%) made reference to the fact that they felt the presence of older and more frail residents contradicted what they had been “sold” when they moved to the community.

This is advertised as independent living for the active over 55-year-olds. That’s the advert, you will see that … so whereas the average age here might have been 75, it’s now over 80, and we feel that we are in an “old people’s home” rather than “active, independent living” …. And we are not the only people, we’ve had a lot of arguments with [management company] about this. (Roy, 72, UK)

We don’t want to be tripping over Zimmer frames the whole time and it’s a bit depressing in a way to see these people who really ought to be in a nursing home or in care. (John, 73, UK)

Some (10%) objected to the burden of satisfying “care responsibilities” to help those more dependent and frailer.

We’ve had to tell them we are good neighbours, we are not carers. I’m not here to be a carer. We didn’t sign up to look after people, we signed up to have an independent life. We want to be in a community of people who are active and independent. We don’t want to have people who depend upon us for their daily lives. (Roy, 72, UK)

#### The other side of the coin

The smaller percentage of residents in the “prompted by dependency group” (10%), who had moved in to seek support and assistance for increased dependency needs, articulated lived experiences of being “othered” in the community that often reflected a sense of isolation and exclusion (van Dyk, 2016). As mentioned above, Peter and his wife, Sue, had moved into their retirement community on the understanding that it would provide them with a sense of support and assistance as Sue’s dementia became increasingly challenging for them.

Well, she’s tried the book club here and she gets so … how best describe it? Frustrated, because she can’t complete a sentence, frustrated, because she hasn’t been able to read the books because the font size is too small, she has to have a relatively large font size to be able to read, it has to be a light-ish book because otherwise it’s too heavy for her … and so on.

But my point is they don’t really take it on board in the group and provide the support I think she needs. And, in some ways, now, I just feel she’s a bit like a leper really—because no one actually wants to get close to her here. (Peter, 78, UK)

Fundamental to such exclusion was a perceived lack of understanding (from other residents and management/staff) of the “prompted by dependency group” residents’ needs in the community.

We’ve been disappointed in that sense. The care isn’t there that we thought was going to be there. I don’t think people here understand what dementia is all about. (Peter, 78, UK)

### Developing a Sense of Community

Despite the conflict of needs and the division captured above, some residents (35%) clearly articulated a sense of community and placed value on both supporting those more vulnerable than themselves and on being supported by other residents. When continuing to live in a community where dependency and frailty increasingly become norms, residents (15%) like Milly seemed to value and genuinely take pride in supporting and providing for other members of the retirement village and clearly played a role in fostering and enhancing a sense of care and community.

When my late husband, Doug, lived here [in the village] too, he got very, very involved. Anybody around the place that had a problem, Doug would fix it. Sometimes I’d think, “Where is he? I wish he would come in and have his lunch.” He’d be somewhere talking to somebody or fixing something for somebody!

He [Doug] said, “I wonder what they are doing for the turn of the century.” I said, “I don’t know.” Doug said, “Well we are going to have a barbecue,” so we started it. Anyway, I don’t know how many people we had but it was chockers, everybody came … it was beautiful. So, we started doing it in the village every month, a barbecue, and we did it for 10 years! (Milly, 88, Australia)

We found that such a sense of community was closely connected to the lived experience and expectations regarding *growing older together*. The significance of sharing lives and nurturing relationships in everyday community living was clearly evidenced in the United Kingdom and Australian villages, but sometimes in distinctive ways. In the UK villages, the residents had lived in their villages on average for less than 3 years. While admitting a sense of community was yet to be fostered, they (15%) believed that aging in the community was key to nurture intimacy and belongingness as a means of defending from impending aging-related challenges.

At the moment, living here, we are more the other side of things, we are more the people that the neighbours say, “Can you help with this or do that?” “Take me somewhere or do that.” But I think it would work the other way round. I think maybe if we both get older and more connected here we would possibly become dependent on very close friends here. (Ralph, 72, UK)

On the other hand, the residents (20%) in Australia had a significantly longer time aging together with others in the villages for an average of 8 years; a few (10%) like Milly, 88, had even lived in their community for over 15 years. As such, an ongoing sense of care and community was captured either within a small group or by a community as a whole.

Emma: In the area where we lived [pre-retirement community] … we didn’t know so many people around us … but here [in the village] I can say I know everybody here.

Interviewer: Does it feel like a community?

Emma: Yes, it does. Yes. (Emma, 87, Australia)

## Discussion

In line with Wiles et al.’s (2012) argument, we took the position that older people living in retirement villages are a far from homogeneous group and likely have diverse and often contrasting ideas about what they need and hope to find in relation to independence, dependence, and autonomy (Hillcoat-Nallétamby, 2014). Our data revealed significant and meaningful differences in relation to what older people may be seeking in relation to independence and dependence when they choose to move to a retirement community. A significant group of participants reflected a typically younger subset, living in a “predependency” phase, yet to experience serious decline in health or significant increases in physical, social, or emotional dependency. Such participants clearly viewed the retirement community as a space in which they sought to retain a sense of “active living,” where they could feel fully independent, active, and “prolong midlife” (McHugh, 2000). They expressed a feeling of security in the idea that the retirement community offered them a “future proofing” against increased dependency or loss as they aged.

In contrast, a smaller subset of participants had clearly been “prompted” by increased dependency needs to move to a retirement community and articulated very different needs and ideas around dependence and independence. For such residents, the retirement community reflected a space where they hoped that their increased dependency needs (which could relate to physical or cognitive health decline, disability, or social and emotional deficits) would be understood, accepted, and supported, so that they might retain and protect relative independence and autonomy in the face of significant aging-related challenges.

To some extent, this variation in needs and narratives around independence and dependence may reflect the lack of consensus around what retirement villages are (and are not), raised earlier in the article (Glass & Skinner, 2013). In our study, older people with very different ideas of what independence/dependence meant (for them), identified, bought into, and were sold different promises about the same villages. Beyond the basic parameters of being retired, having geographic boundaries, offering shared activities and facilities, and a sense of “autonomy” and “security” (however defined), the retirement villages in our study frequently offered different (and sometimes incompatible) narratives and meanings to different people.

Our data clearly revealed narratives around “sameness” (us) and “difference” (them) that were tied to the degree of independence and/or dependence older people embodied. Jönson (2013) has discussed how the *otherness* of older people is revealed in language that signifies “difference,” “comparison,” and “categorization” and is used by members of in-groups to discriminate against members of “out-groups.” In our study, there was a clear use of language connected to extreme old age (“the average age here is nearly 80. That’s not active retired, is it? Somebody moved in who was 94?!”) and a need for care and dependency and/or deteriorating capabilities (“We don’t want to be tripping over Zimmer frames the whole time … it’s a bit depressing in a way to see these people who really ought to be in a nursing home”) that created a sense of “us” (the active, independent residents) and “them” (the old, frail, needy, and dependent residents) in the retirement villages.

The construction of such otherness around themes of dependency and independence within retirement villages is potentially problematic for several reasons. First, it risks creating a clear sense of feeling discriminated against or excluded in members of the outgroup (e.g., “I just feel she’s a bit like a leper really—because no one actually wants to get close to her here”). Second, perhaps retirement villages cannot be “all things to all people.” It is, for example, contradictory to suggest that villages can fully support, accept, include, and accommodate older people who move in with significant dependency needs, while simultaneously providing an environment that satisfies the wishes of those who might prefer to live in a community that embodies “active independence” or “prolonged midlife.” Third, as Jönson (2013) argued, there is a paradox in the othering of more frail, vulnerable, and dependent older people that may be irrational because “according to all human experience, we inevitably have to face it …. Some kind of misunderstanding must be the cause: why else would we want to dispose of a percentage of the population that we will be part of in the future?” (p. 199).

It is important for developers and providers of retirement villages to recognize their role in constructing conflicts and collision in relation to narratives of independence and dependence for older people. Ageism may be amplified by retirement villages that strongly promote an image of “active independent living.” McHugh (2000) has argued that the “ageless self” and the “prolongation of midlife” have become “the leitmotif of contemporary society, conveying little about change and what it really means to grow old” (p. 103). He further argued that such ageist attitudes and societal values are firmly embedded in the identities of certain retirement villages and, thus, such villages play a role in “selling” and subsequently “reinforcing” an unrealistic and potentially discriminatory idea of growing old.

Our data also raised the ethical issue of retirement villages “selling different ideals to different people.” The tensions this created when such residents lived alongside each other were clear to see in our data set, and careful consideration is required in relation to what older people are “sold” when they move to a retirement community.

It should be noted that some residents deeply valued supporting those more dependent than themselves, contributing to a sense of inclusivity within the community. These residents actively sought bonds and solidarity with other residents, aiming to cope with aging in a more communal manner. Lawrence and Schigelone (2002) have raised the possibility that age-related stressors often thought of as “individual” in nature can be shared, expressed, and coped with “communally.” It is possible that retirement villages offer the prospect of communally coping with increased dependency and vulnerability associated with aging. In our study, this was particularly clearly demonstrated among some Australian residents who had lived together for a considerable time. Although the duration of time in a place is not the only determinant, the experiences of growing older together, over time, could significantly shape residents’ experiences of aging and increased dependency in a positive manner.

Some older people in our study, who were part of the “predependency” group, implicitly understood that those in the “prompted-by-dependency” or “became-more-dependent-in-place” groups may well be a reflection of their future selves and, as such, they were more able to accept and acknowledge these older people as part of the community. Given the unrealistic ideal of an eternal midlife and the fact that people with higher dependency needs are simply further along a pathway that most will (in different ways) likely follow at some point in their lives, it makes sense for retirement villages to devote time and resources to creating a community where, rather than “othering,” people seek to better understand, support, and include others who may simply be embodiments of their future selves (Jönson, 2013).

In conclusion, McHugh (2000) has argued that retirement communities reflect “societal scripts in successful aging … defined as much by the absent image—old, poor folks … as by the image presented: handsome, healthy, comfortably middle-class ‘seniors’, busily filling sun-filled days” (p. 113). As Laws (1995) suggested over 25 years ago, the project for future retirement villages will necessarily involve moving beyond retirement communities that simply reinforce this script. Our data also raise questions already outlined in the literature (Glass & Skinner, 2013; Wiles et al., 2012) related to whether communities designed for “older people” as a homogeneous category are a desirable model if they (a) inadequately recognize the diverse and conflicting needs of an extremely wide range of people and (b) are often so vaguely defined that they appear to offer “all things to all people.”
